# Canalization of Gene Expression in the *Drosophila* Blastoderm by Gap Gene Cross Regulation

**DOI:** 10.1371/journal.pbio.1000049

**Published:** 2009-03-10

**Authors:** Manu, Svetlana Surkova, Alexander V Spirov, Vitaly V Gursky, Hilde Janssens, Ah-Ram Kim, Ovidiu Radulescu, Carlos E Vanario-Alonso, David H Sharp, Maria Samsonova, John Reinitz

**Affiliations:** 1 Department of Applied Mathematics and Statistics, and Center for Developmental Genetics, Stony Brook University, Stony Brook, New York, United States of America; 2 Department of Computational Biology, Center for Advanced Studies, St. Petersburg State Polytechnical University, St. Petersburg, Russia; 3 Theoretical Department, The Ioffe Physico-Technical Institute of the Russian Academy of Sciences, St. Petersburg, Russia; 4 EMBL/CRG Research Unit in Systems Biology, CRG–Centre de Regulació Genòmica, Barcelona, Spain; 5 Institute of Mathematical Research of Rennes, University of Rennes 1, Rennes, France; 6 Theoretical Division, Los Alamos National Laboratory, Los Alamos, New Mexico, United States of America; Cambridge University, United Kingdom

## Abstract

Developing embryos exhibit a robust capability to reduce phenotypic variations that occur naturally or as a result of experimental manipulation. This reduction in variation occurs by an epigenetic mechanism called canalization, a phenomenon which has resisted understanding because of a lack of necessary molecular data and of appropriate gene regulation models. In recent years, quantitative gene expression data have become available for the segment determination process in the *Drosophila* blastoderm, revealing a specific instance of canalization. These data show that the variation of the zygotic segmentation gene expression patterns is markedly reduced compared to earlier levels by the time gastrulation begins, and this variation is significantly lower than the variation of the maternal protein gradient Bicoid. We used a predictive dynamical model of gene regulation to study the effect of Bicoid variation on the downstream gap genes. The model correctly predicts the reduced variation of the gap gene expression patterns and allows the characterization of the canalizing mechanism. We show that the canalization is the result of specific regulatory interactions among the zygotic gap genes. We demonstrate the validity of this explanation by showing that variation is increased in embryos mutant for two gap genes, *Krüppel* and *knirps*, disproving competing proposals that canalization is due to an undiscovered morphogen, or that it does not take place at all. In an accompanying article in *PLoS Computational Biology* (doi:10.1371/journal.pcbi.1000303), we show that cross regulation between the gap genes causes their expression to approach dynamical attractors, reducing initial variation and providing a robust output. These results demonstrate that the Bicoid gradient is not sufficient to produce gap gene borders having the low variance observed, and instead this low variance is generated by gap gene cross regulation. More generally, we show that the complex multigenic phenomenon of canalization can be understood at a quantitative and predictive level by the application of a precise dynamical model.

## Author Summary

Animals have an astonishing ability to develop reliably in spite of variable conditions during embryogenesis. More than 60 years ago, it was proposed that this property of development, called canalization, results from genetic interactions that adjust biochemical reactions so as to bring about reliable outcomes. Since then, a great deal of progress has been made in understanding the buffering of genotypic and environmental variation, and individual mutations that reveal variation have been identified. However, the mechanisms by which genetic interactions produce canalization are not yet well understood, because this requires molecular data on multiple developmental determinants and models that correctly predict complex interactions. We make use of gene expression data at both high spatial and temporal resolution for the gap genes involved in the segmentation of *Drosophila*. We also apply a mathematical model to show that cross regulation among the gap genes is responsible for canalization in this system. Furthermore, the model predicted specific interactions that cause canalization, and the prediction was validated experimentally. Our results show that groups of genes can act on one another to reduce variation and highlights the importance of genetic networks in generating robust development.

## Abstract 3

During*Drosophila* development, the expression patterns of gap genes are much less variable than the Bicoid morphogen gradient. Modeling and experiments show that this specific instance of canalization or developmental robustness occurs by gap gene cross regulation.

## Introduction

C. H. Waddington inferred developmental canalization of gene expression by noting that differentiation leads to the formation of discrete types of tissue, rather than a continuous blend, and that genotypic and environmental variation is suppressed at the phenotypic level in wild-type but not mutant organisms. These points led him to state that “developmental reactions, *as they occur in organisms submitted to natural selection*, are in general canalized. That is to say, they are adjusted so as to bring about one definite end-result regardless of minor variations in conditions during the course of the reaction” (p. 563 in [1]). These adjustments manifest themselves as a reduction in the variation of phenotypes. In this article, we make a detailed experimental and theoretical study of the canalization process in the gap gene system of *Drosophila*. Interestingly, this investigation puts us in contact with a number of recent investigations of variation in the gap gene system as we now explain.

Among contemporary geneticists and evolutionary biologists, the buffering of phenotypic variation to underlying genotypic variation in wild type is well known, and recent experimental studies have identified individual genes that are responsible for this aspect of canalization [2,3]. Theoretical studies have demonstrated that this phenotypic buffering is an intrinsic consequence of the adjustments of developmental trajectories postulated by Waddington [4], but direct evidence of developmental canalization was still lacking. Direct evidence of developmental canalization must meet two requirements. First, the system must show variation between individuals that decreases significantly over the course of development. Second, this decrease in variation must be demonstrated to be an inherent property of the system under study, rather than being imposed from a different part or from outside the organism. The first requirement implies the need for data on the dynamics of developmental determinants, some or all of which will be molecular. The demonstration that reduction in variance of a developmental system is inherent to that system requires precise understanding of the consequences of the interaction of many developmental determinants. Understanding such consequences requires a quantitative model.

The segmentation system of Drosophila melanogaster [5,6] is highly suitable for studies of canalization because it is already known that the first of the above requirements, decrease in phenotypic variability over time, is satisfied. In work reported elsewhere [6], we performed a quantitative analysis of the expression of segmentation genes expressed from the maternal genome only (*bicoid*), the maternal and zygotic genomes (*caudal* and *hunchback*), and the zygotic genome (*Krüppel*, *giant*, *knirps*, *tailless*, *fushi tarazu*, *even skipped*, *runt*, *hairy*, *odd skipped*, *paired*, and *sloppy paired*). These expression data have cellular resolution in space and about 6.5-min resolution in time, and comprise either the expression levels of *even skipped* (*eve*) and two other genes in individual embryos or the integrated averaged expression of all 14 protein gene products. These data show that segmentation gene expression is highly variable among individual embryos in cleavage cycle 13 and the early part of cycle 14. There is extensive variation in expression levels, locations of domain borders, and the time and order of the appearance of individual domains. However, the variation in the expression patterns reduces over time and is significantly lower at the onset of gastrulation than at earlier times.

In this article, we restrict our attention to a particularly important class of phenomena concerning the variation in the location of the boundaries of zygotic gene expression domains. These boundaries shift when maternal gradients are perturbed, and hence are at least in part under the control of these gradients [7,8]. Nevertheless, the variation in the boundary positions of gap and pair-rule expression domains is much lower than the variation in the maternal gradient of Bicoid (Bcd) protein [6,9,10]. Under the simplest model of specification, expression borders would form at a fixed threshold of its concentration [11]. The variation of the Bcd gradient can be measured in terms of the range (ρ_Bcd_) or standard deviation (σ_Bcd_) of the position (*x*_Bcd_) where it crosses the threshold concentration. In our data σ_Bcd_ = 4.6% egg length (EL), whereas gap gene domain border positions have σ ∼ 1% EL [6]. Pair-rule stripe border positions also have similar low variation [6] compared to the Bcd threshold position.

Of the large number of gene expression borders that have lesser variation than Bcd, the posterior border of the anterior *hunchback* (*hb*) domain has received intense scrutiny [9,12–18]. Gregor and colleagues [16] measured the absolute concentration of Bcd in the nuclei at the *hb* border using a Bcd-GFP fusion rescue construct. This measurement led the authors to two important but mutually contradictory conclusions. First, using a result from bacterial chemotaxis theory [19], it was shown that noise due to small number of Bcd molecules is high enough so that the *hb* border cannot be accurately specified by Bcd alone. Second, by measuring Bcd profiles from several live embryos in parallel, it was found that σ_Bcd_ ∼ 2% EL. The amount of variance reduction implied by this value of σ_Bcd_ is significantly smaller than the estimates from fixed-tissue experiments [6,9], presumably because the GFP measurements do not have scaling error introduced in the setting of microscope gain in separate fixed-tissue experiments. Under the assumption that Bcd is the only regulator of *hb*, it was argued that these results can be reconciled if spatial averaging from the diffusion of Hb molecules is taken into account, and the authors draw the conclusion that Bcd alone is sufficient to specify the *hb* border accurately.

**Figure 1:**
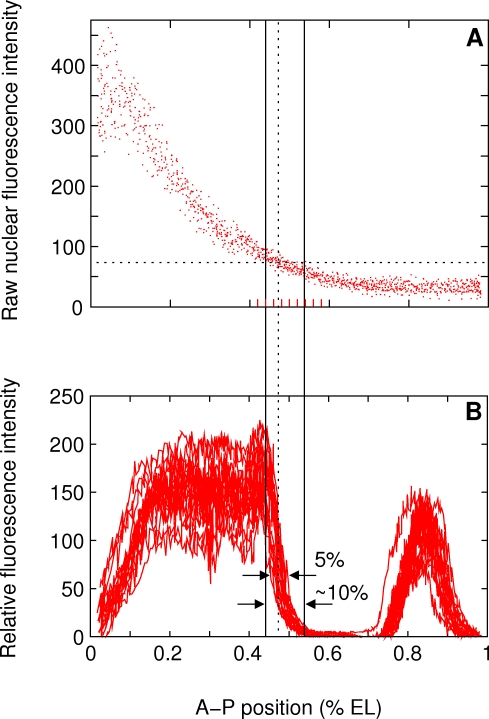
Comparison of In Vivo Bcd Variability with Hb Variability (A) Fifteen Bcd-GFP concentration profiles replotted from the data of Gregor and colleagues (Figure 5A in [16]). (B) Eighteen Hb profiles 3 min before gastrulation (time class T8, see Table 1) from FlyEx [6]. The dotted vertical line is at the average position of *hb* border. The dotted horizontal line passes through the point where the dotted vertical line crosses the middle of the Bcd scatter. The solid black lines delineate the horizontal spread of Bcd. The red tick marks have been placed uniformly using the graphics program Xfig.

The assumption that *hb* is under the sole control of Bcd is incorrect, because its border position changes in embryos mutant for *giant* (*gt*) [9], *Krüppel* (*Kr*) [20], *Krüppel*;*knirps* (*Kr*;*kni*) [20], and in embryos lacking Nanos [9]. Apart from the average position, the variation of the position is also increased in embryos lacking chromosome arm 3L or Nanos to ∼2% EL and ∼1.6% EL respectively [9], close to the Bcd variation measured in live embryos. The Bcd-dependent response of a fragment of the *hb* promoter has variation close to Bcd variation (see Figure 4 in [21]) (ρ_Bcd_ ∼ 9% EL; compare with Figure 1A) and higher than the variation of endogenous *hb* (Figure 1B). All these experiments suggest that endogenous *hb* expression has lower variation than Bcd, which is increased to the level of Bcd variation when other inputs to *hb* are removed.

**Table 1:**
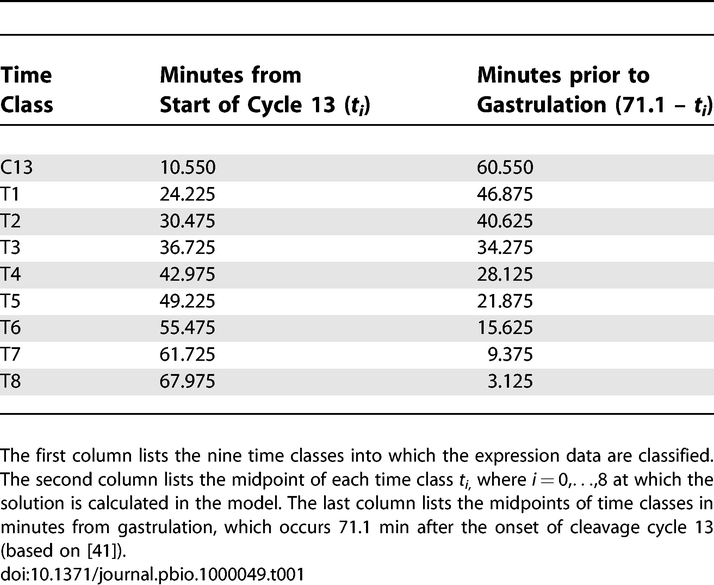
Time Classes

**Figure 2:**
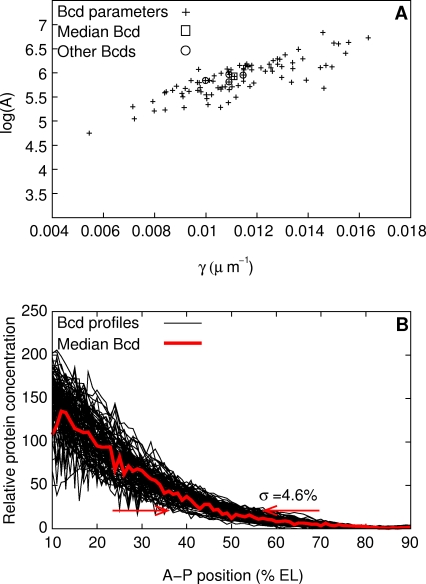
Selection of a Representative Bcd Profile (A) Scatter plot of log amplitude (log *A*) and slope (γ) of 88 Bcd profiles from cycle 13 embryos. The boxed profile and the circled profiles were investigated further; unless explicitly mentioned, the analysis in this paper uses the boxed (median) profile. (B) An overlay of all 88 Bcd profiles used in the simulations. The median profile is highlighted in red. The threshold concentration at which the Hb border forms in the gene circuit was determined from the median profile. The positions at which these 88 profiles cross that threshold has a range of 20.6% EL, and a standard deviation (γ) of 4.6% EL.

To make the relationship between Bcd variation and *hb* border variation clear, we have plotted the data of Gregor et al. and compare it with fixed-tissue Hb data (Figure 1). The *hb* border is steep, and its position can be measured unambiguously in fixed tissue since it is not sensitive to scaling variation, unlike a Bcd threshold concentration. This comparison shows that the Bcd threshold position at which the *hb* border forms is about twice as variable as the border position. It is important to note that the *hb* border is just one among many that have low positional variance. As an example, consider the anterior border of the posterior *kni* domain, which is activated by Bcd [22]. This border is located ∼10% EL posterior to the *hb* border and has a positional variance of ∼1% EL [6]. At this position, the Bcd-GFP data have a variance of ∼4% EL (see Figure 5C in [16]), which is much larger than that of the *kni* border. This example clearly contradicts a picture of positional specification in which Bcd specifies all of its targets accurately.

Like *hb*, *kni* is not regulated by Bcd alone, but also by Caudal (Cad) [22,23], Hb [20,24], and Gt [25]. The examples of the *hb* and *kni* borders show that it is not possible to explain the variance of a single expression border position in isolation. It is necessary to take into account all the genetic interactions required for generating the expression patterns over space and time. Gene circuits [26,27] are dynamical models that can accurately reproduce observed gene expression patterns by reconstituting the required set of genetic interactions in silico. In the gene circuit method, (1) a gene circuit model, in which one real number characterizes the regulatory effect of one gene on another, is (2) fit to quantitative gene expression data by (3) parallel Lam simulated annealing [28,29] or some other nonlinear optimization method [30–32], and finally (4) biological conclusions are obtained. This method has been successfully used to analyze both the pair-rule and gap systems [27,33–36] and performed better than other models in a comparative study [31].

In what follows, we briefly describe the gene circuit used in the Results section “Gap Gene Circuits”; a full description is provided in Protocol S1 (Section S1). We then demonstrate in the Results section “Simulation of Bcd Variation and Size Variation” that a gene circuit model for the gap genes *hb*, *Kr*, *gt*, and *kni* under maternal control and including mutual gap–gap interactions is sufficient to explain the reduction in the variance of gap gene domain border positions, showing that it is not necessary to postulate an undiscovered gradient [12,14,15] or active transport [13] to explain this property. In the Results section “Variance Reduction by Gap Gene Cross Regulation and Experimental Verification,” we characterize the regulatory interactions underlying variance reduction. This analysis shows that the reduction in variance of the *hb* border is a consequence of repression by Kr and Kni, and hence predicts an increase in variance when these factors are removed. We present new experimental data from *Kr*;*kni* double mutant embryos that validate this prediction and show the correctness of the gene circuit approach. The validation of the gene circuit approach shows that the gap gene system also satisfies the second requirement for demonstrating canalization, which is that variance reduction arises from intrinsic properties of the system.

## Results

### Gap Gene Circuits

We constructed a gene circuit that models the expression patterns of the gap genes *hb*, *Kr*, *gt*, and *kni* in a region of the blastoderm extending from 35% EL to 92% EL along the anteroposterior (A–P) axis. The protein products of these genes are transcription factors [37–40] and localize to nuclei. The model computes the time course of nuclear protein concentrations in a time interval beginning at the start of cleavage cycle 13 and ending at the onset of gastrulation [41] (Figure S2 in Protocol S1). The initial conditions were specified using cleavage cycle 12 gene expression data [6] (Section S1 in Protocol S1). The 13th nuclear division occurs in the duration of the model (Figure S2 in Protocol S1) after which the nuclei in the modeled region are divided, and daughter nuclei inherit the state of the mother nucleus.

The gene circuit equations model the processes of protein synthesis, degradation, and diffusion (Section S1.1 in Protocol S1). During the mitosis preceding the 13th nuclear division, the protein concentrations are governed only by diffusion and degradation as synthesis shuts down [42]. During interphase, the synthesis rate of a protein depends on the regulation of the gene by the other gap proteins, the maternal gradient Bcd, and the upstream regulators Cad and Tailless (Tll). The regulatory effect of a protein is characterized by the product of its concentration and its regulatory strength. The concentration varies from nucleus to nucleus, but the regulatory strength, a parameter of the model, is invariant throughout the embryo, reflecting the fact that the zygotic genome is the same in each blastoderm nucleus. The sum of all the regulatory terms for a gene, called the total regulatory input (*u*), determines its synthesis rate through the sigmoidal regulation-expression function 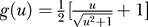 (Figure S1 in Protocol S1).

To fully specify the model the concentrations of Bcd, Cad and Tll must be provided. The concentrations of Cad and Tll were provided by the interpolation of average data [6] in time (Section S1.2 in Protocol S1). A representative profile of Bcd was provided as follows.

The Bcd gradient is essentially stationary during cleavage cycles 13 and 14 [6,43,44], and hence, its concentration *v* ^Bcd^ is assumed to be constant in time. It is known through antibody studies [6,9,12,43] and a recent GFP-Bcd [44] study that the Bcd profile is an exponential function of A–P position *x*, so that

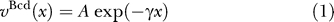
The arithmetic mean of exponential curves is not exponential. Thus, it is not possible to generate a representative Bcd profile by taking an arithmetic average over embryos. Instead of averaging, a representative individual Bcd profile *v* ^Bcd^(*x*) was chosen in the following manner. We obtained data from 88 cycle 13 embryos immunostained for Bcd and removed the background signal from the Bcd profiles as described [45]. Taking the logarithm of Equation 1, we get

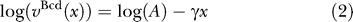
The background-removed profiles were then fit by linear least-squares to Equation 2. This procedure yielded two parameters for each profile, γ (length scale), and *A* (concentration scale). Figure 2A is a scatter plot of log *A* with γ. A profile (“median” profile) was chosen such that its parameters lie in the middle of the scatter plot (Figure 2A). Since this study concerns only embryo-to-embryo variation and not nucleus-to-nucleus noise, the exponential fit of the profile was used in the model. Using background-removed profiles directly in the model yields circuits with the same properties as those of circuits with exponential fits (See section “Simulation of Bcd Variation and Size Variation” below and Table S4 in Protocol S1).

To compare with quantitative gene expression data [6], we calculate the solution of the model at multiple time points. The data are classified into time classes to give nine time points for comparison (see Table 1 and Figure S2 in Protocol S1).

### Simulation of Bcd Variation and Size Variation

We obtained specific gene circuits by performing a least-squares fit of the model to gene expression data from nine time points (see Methods). We used a median Bcd profile from an individual embryo as described in the previous section, together with averaged expression profiles from the other genes considered. Twenty-three circuits were obtained with almost identical network topology (Table S3 in Protocol S1) and expression patterns. One of these circuits was chosen for further study. Its parameters are given in Protocol S1 (Tables S1 and S2). The circuit's gap gene patterns (Figure S4A and S4B in Protocol S1) are consistent with data except for two minor defects (Section S1.3 in Protocol S1) and the circuit has the same network topology that was obtained previously [31,35,36].

#### Bcd variation.

We simulated the effects of embryo-to-embryo variation by running an ensemble of 88 simulations of the chosen circuit such that each simulation used a Bcd profile from a distinct individual embryo (Figure 2B). All parameters of the circuit were kept fixed, and only the Bcd input to the circuit was varied across the ensemble. The positional variance of the gap gene borders was compared with that of Bcd profiles. The position of a gap gene domain's border was calculated as the point where the concentration of the gene's protein product was half its maximum level in that domain (see Methods). The positional variance of a border is the standard deviation of its position in the 88 simulations. The positional variance of the family of Bcd profiles is the standard deviation (σ_Bcd_) of the position (*x*_Bcd_) at which each profile crosses a threshold concentration. The Bcd threshold for a particular gap gene border is the Bcd concentration at which the border forms in the circuit with the median Bcd.

**Figure 3:**
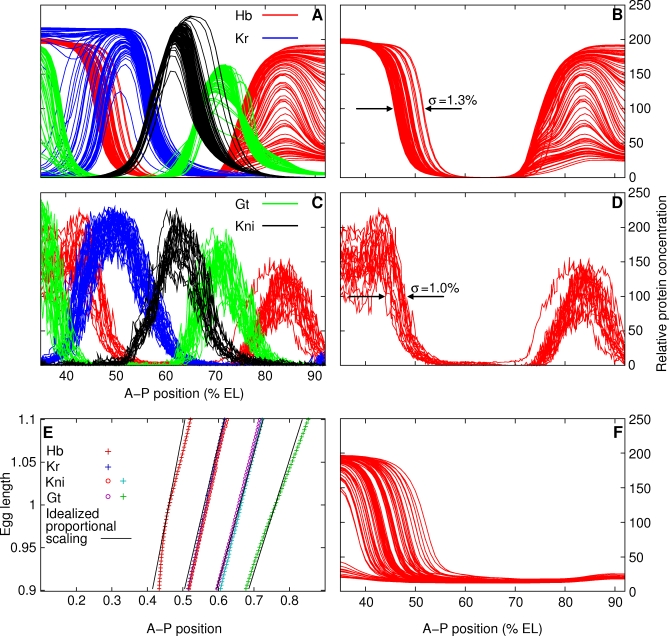
Gene Circuits Correctly Predict the Variation of Six Gap Gene Domain Borders (A) Modeled expression patterns of *hb*, *Kr*, *gt*, and *kni* in time class T8 (see Table 1) in simulations of the gene circuit using Bcd profiles from 88 individual embryos; compare with (C). (B) *hb* patterns produced by the model are shown separately. The position of the posterior border of the anterior *hb* domain has σ_Hb_= 1.3% EL and ρ_Hb_ = 5.6% EL. (C) Gap gene expression data from 83 time class T8 wild-type embryos. There are 15 expression profiles for *hb*, 30 for *Kr*, 18 for *gt*, and 15 for *kni*. (D) σ_Hb_ = 1.0% EL and ρ_Hb_ = 3.6% EL in data. (E) Simulation of size variation. The egg length was varied from 0.9 to 1.1 to simulate a 20% range of egg lengths. The *x*-axis is the absolute position (relative position × length) of the gap gene borders. The border positions of the six gap gene domain borders that have low variance under Bcd variation (A) are plotted as points. The solid black lines show idealized proportional scaling of border positions with egg length. (F) In silico control experiment of simulating Bcd variation with gap gene cross regulation of *hb* turned off. The Bcd profiles used were the same as the ones used to produce (A) and (B). Fourteen borders are located anterior to 35% EL and are not visible in the region shown. σ_Hb_ = 4.3% EL and ρ_Hb_ = 19.6% EL.

The model correctly predicts the positional variance of six gap gene borders: the posterior border of the anterior *hb* domain, the posterior border of the central *Kr* domain, both borders of the posterior *kni* domain, and both borders of the posterior *gt* domain (see Figure 3A and 3C and Table 2). For example, the posterior border of the anterior *hb* domain has a standard deviation of 1.3% EL in the simulations and 1.0% EL in data (Figure 3B and 3D), both of which are much less than the standard deviation of the Bcd threshold position, 4.6% EL (Figure 2B). To further confirm that the gene circuit was reducing Bcd variation, we performed the in silico control experiment of turning off gap gene cross regulation of *hb* in the model. In this simulation, the variation of the posterior border of the anterior *hb* domain increased to 4.3% EL, close to the level of variation in the Bcd threshold position (Figure 3F).

**Figure 4:**
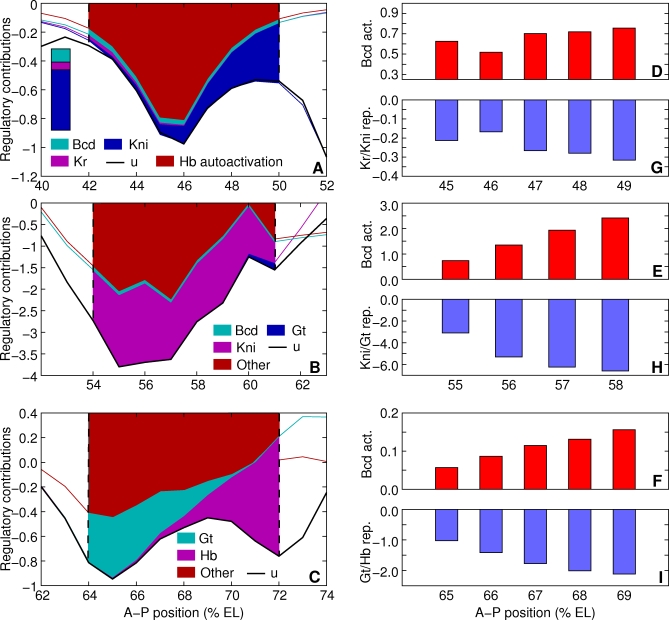
Regulatory Analysis of the Posterior Borders of the Anterior *hb*, the Central *Kr*, and the Posterior *kni* Domains (A–C) Analysis of the chosen gene circuit (see Results section “Simulation of Bcd Variation and Size Variation”) with the median Bcd profile (Figure 2B). Dashed vertical lines demarcate a border, and correspond to positions where the expression is at 90% maximum and at 10% maximum. The solid black line is the spatial derivative of the total regulatory input to the gene *u^a^* (see Section S3 in Protocol S1). The area above the black line is the total change in total regulatory input *u^a^* that causes the border to form. The colored areas correspond to the contributions to the change in total input by different regulators of the gene. The regulatory inputs that cannot set a border are shown in red (see Section S3 in Protocol S1 for details), and are not included in the analysis. A regulatory contribution can be from an activator or a repressor depending on the sign of the regulatory parameter (see Table S1 in Protocol S1 for values). (A) The *hb* border forms because of the regulatory contributions of Bcd activation, Kr repression, and Kni repression. The contribution of Hb autoactivation, shown in red, does not set the border but merely sharpens it [9,36]. The colored bar inset shows the relative contributions of Bcd activation, Kr repression, and Kni repression to *hb*, showing that the repressive contribution is significant compared to the activating one. (B) The *Kr* border is set by Bcd activation and repression from Kni and Gt. (C) The *kni* border is set by repression from Gt and Hb. (D–I) The effects of simulated Bcd variation on the regulation of gap gene borders. (D–F) The average Bcd activation at a border in 88 simulations (with different Bcd profiles) pooled into 1% EL bins according to border position. (G–I) The average repression levels at a border in 88 simulations pooled into 1% EL bins according to border position. The repressors were identified in the regulatory analysis (A–C). The *x-*axis shows bin position. The analysis was performed on model output in time class T8.

**Figure 5:**
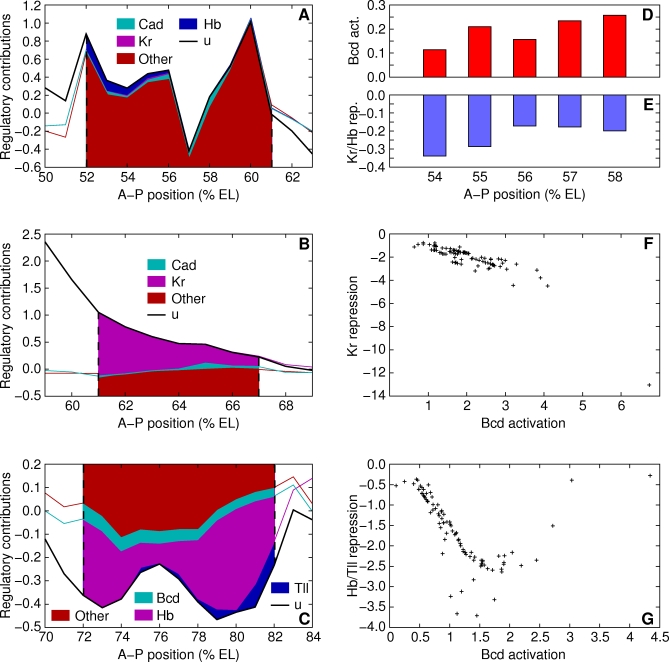
Regulatory Analysis of the Anterior Border of the Posterior *kni* Domain and Both Borders of the Posterior *gt* Domain (A–C) Analysis of the chosen gene circuit (see Results section “Simulation of Bcd Variation and Size Variation”) with the median Bcd profile (Figure 2B). The graphical regulatory analysis is described in the caption of Figure 4. (A) The *kni* border forms because of the regulatory contributions of Cad activation, Kr repression, and Hb repression. (B) The anterior *gt* border is set by Cad activation and repression from Kr. (C) The posterior *gt* border is set by Bcd activation and repression from Hb and Tll. (D–G) The effects of simulated Bcd variation on the regulation of gap gene borders. (D) The average Bcd activation at the *kni* border in 88 simulations (with different Bcd profiles) pooled into 1% EL bins according to border position. (E) The average repression levels at the *kni* border in 88 simulations pooled into 1% EL bins according to border position. (F) Scatter plot of the Bcd activation and Kr repression at the anterior *gt* border in each of the 88 simulations. (G) Scatter plot of the Bcd activation and Hb/Tll repression at the posterior *gt* border in each of the 88 simulations. The repressors were identified in the regulatory analysis (A–C). The *x-*axis shows bin position. The analysis was performed on model output in time class T8.

**Table 2:**
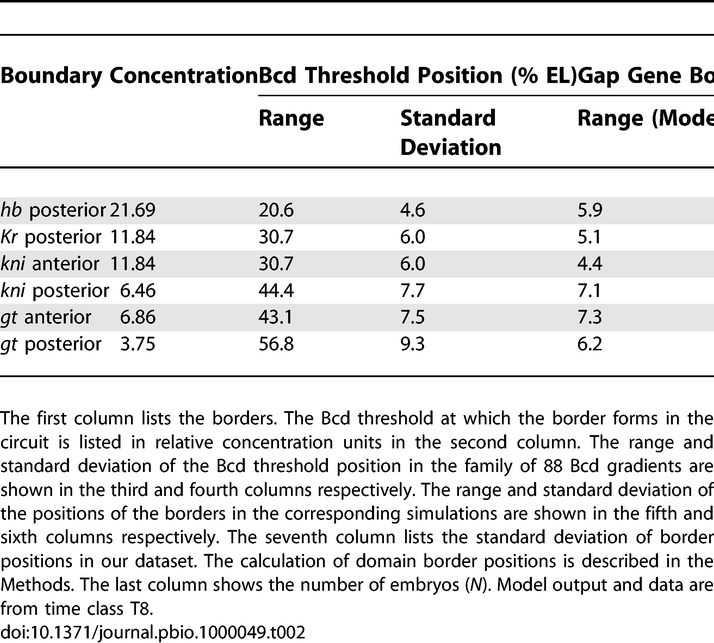
Comparison of the Positional Variance of Six Gap Gene Borders with the Positional Variance of Bcd

Of the 23 circuits that had consistent network topology, 15 showed low positional variation for these six borders. The ability of the gene circuit to filter out Bcd's positional variation does not depend on the particular Bcd profile used to fit the circuit. Circuits produced with other profiles from the middle of the parameter scatter (black circles in Figure 2A) also showed accuracy when subjected to Bcd variation (see Table S4 in Protocol S1). The gene circuit has this filtration property irrespective of whether smoothed or unsmoothed profiles are used to simulate Bcd variation (Table S4 in Protocol S1).

Four borders show large variation: the posterior border of the third *gt* domain, the anterior border of the central *Kr* domain, and both the borders of the posterior *hb* domain (see Figure 3A). All these borders are at the edges of the modeled region, where the model is missing key regulators. In the anterior, the head gap genes *orthodenticle*, *empty spiracles*, and *buttonhead* [46–48] are excluded. They are known to influence the anterior pattern of trunk gap genes [25], and respond to Bcd in a concentration-dependent manner [49]. In the posterior terminal region *huckebein* (*hkb*) [50–52] has been omitted from the model.

#### Size variation.

*Drosophila* eggs vary in length by 10–20%, and different wild lines may have differing mean egg lengths [53]. Although the location of *eve* stripes [53] and the posterior border of the anterior *hb* domain scale with egg length, the Bcd gradient and hence its threshold positions do not [9]. To explain this scaling property, an as-yet-undiscovered gradient has been proposed [12,14]. These proposals specifically considered the scaling of the *hb* border, but one theoretical study suggests that the second gradient could scale multiple borders [15]. The hypothetical gradient would be set up from the posterior pole of the embryo and would directly regulate the gap genes or Bcd. The proposed posterior gradient would provide the target with a measure of distance from the posterior pole.

We show here that gap gene circuits exhibit scaling with egg length. In other words, it is not necessary to postulate an undiscovered posterior gradient. *bcd* mRNA is localized at the anterior tip of the embryo [54]. The stationary exponential profile of the Bcd gradient is consistent with the diffusion of the protein from the anterior tip and its degradation by a first-order process, and the gradient has been modeled as such [21,55,56]. Recently, an alternative model, in which degradation is replaced with the reversible trapping of the Bcd protein by the nuclei in the blastoderm, was proposed [57]. Both the trapping model and the diffusion-degradation model do not show scaling of the Bcd gradient with egg length and so we represented the scaling properties of the Bcd gradient using the simpler diffusion-degradation model.

Let *L* denote the length of an embryo and let *z* represent A–P position so that *z* varies between 0 and *L*. Using Equation 1, the concentration of Bcd is given by *v* ^Bcd^(*z*) = *A* exp(–γz), where γ is the characteristic length scale of the profile and *A* is the concentration scale. Let *x* denote the relative position in % EL so that 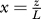 . The Bcd concentration can then be written in terms of *x* as

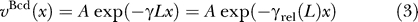
where γ_rel_(*L*) = γ*L* is the length scale in relative position units. Equation 3 may also be arrived at by explicitly rescaling the diffusion-degradation model [21,55,56].

Size variation can be simulated by varying γ_rel_(*L*) in Equation 3. For an embryo with length *L*′ = *L* + Δ*L*

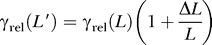
Using the parameters *A* and γ_rel_(*L*) of the median Bcd profile (Figure 2B) we varied 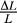 between −0.1 and 0.1 to generate Bcd profiles representing egg length variation of 20%, which corresponds to the range of egg lengths observed in data [9].

We simulated the gap gene expression patterns with these Bcd profiles and the chosen gene circuit (Section S1.3 in Protocol S1). The results of these simulations are shown in Figure 3E. The six gap gene border positions that have reduced variation in the simulations of Bcd variation also scale proportionally with egg length.

### Variance Reduction by Gap Gene Cross Regulation and Experimental Verification

In the previous section, it was shown that the model correctly predicts the positional variance of six borders across a 50% EL region of the embryo. In this section we use the model to characterize the gene interactions responsible for this canalizing behavior of the embryo. This was done in two steps. First, we determined the regulators that set each of the correctly predicted domain borders. The basic idea of this method is that at a border, the relative activation of a gene *g*(*u^a^*) changes from a value close to zero to a value close to one as a result of changes in regulatory input. Those regulatory inputs responsible for changing the state of a gene from off to on can be read from an appropriate graph [33–36] (see Section S3 in Protocol S1 for further details). In the second step, we ascertained how the regulation of a gene at its domain border is affected when subjected to Bcd variation by comparing the strengths of the regulatory inputs at the border position under differing individual Bcd profiles.

The results of the regulatory analysis are shown in Figure 4A–4C for the posterior borders of the *hb* anterior domain, the central *Kr* domain, and the posterior *kni* domain; and in Figure 5A–5C for the anterior border of the posterior *kni* domain and both borders of the posterior *gt* domain. This analysis is consistent with earlier results [31,36]. We briefly summarize the results here; see the captions of these figures and previous studies [31,36] for details. In general, these borders are set up by an activator (either *bcd* or *cad*) and two repressors. There are two tiers of repression. Two pairs of genes with complementary domains in the modeled region, *Kr* and *gt* as well as *kni* and *hb* (Figure S4B in Protocol S1), have strong mutual repression. Genes with overlapping domains in the modeled region, *gt* and *kni* for example, have weak mutual repression. One border, the anterior border of the posterior *gt* domain, is set by a single repressor, *Kr*. This is well supported by the very large anterior shift of this border in *Kr* ^–^ embryos [58].

We studied the effects of varying the Bcd profile in the gap gene circuit as follows. The position of each border was calculated in the 88 simulations using Bcd profiles from individual embryos. With the exception of the borders of the posterior *gt* domain, the simulations were pooled into 1% EL bins according to the position of each border. We then averaged the Bcd activation and gap repression levels at the border position in each group. To understand how the regulation of a border is affected under Bcd variation, we plotted the pooled Bcd activation and gap repression levels together for each border under consideration (Figures 4D–4I and 5D–5G).

The low positional variation of the posterior border of the anterior *hb* domain has been studied extensively [9,12,14–16,59]. Our analysis shows that this border forms by Bcd activation and repression from Kr and Kni (Figure 4A). The results of varying the Bcd profile on the regulation of the *hb* border are presented in Figure 4D and 4G. It shows that both activation and repression levels remain correlated for different positions of the *hb* border, with embryos having *hb* borders in more posterior positions having greater activation by Bcd and greater repression by Kr and Kni. This correlation suggests an epistatic balance between activation and repression at the *hb* border.

**Figure 6:**
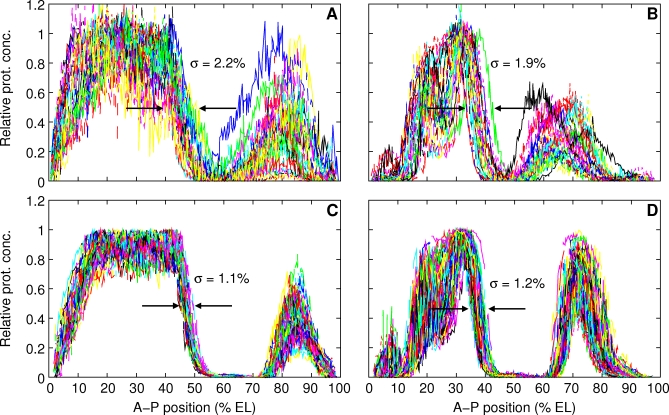
The Control of Positional Variation of the Posterior Border of the Anterior *hb* Domain by Kr and Kni *hb* (A) and *gt* (B) expression profiles in 28 *Kr*;*kni* double mutant embryos from time classes T4–T7 (Table 1); σ_Hb_ = 2.2% EL (standard deviation) and ρ_Hb_ = 9.3% EL (range). The standard deviation of the posterior border of the third *gt* domain is σ_Gt_ = 1.9% EL; ρ_Gt_ = 8.9% EL. *hb* (C) and *gt* (D) expression profiles in wild-type embryos of the same age as in (A) and (B). σ_Hb_ = 1.1% EL and ρ_Hb_ = 5.6% EL (68 embryos). σ_Gt_ = 1.2% EL and ρ_Gt_ = 5.6% EL (92 embryos). Expression patterns are shown normalized against the maximum level (see Methods) in the anterior *hb* (A and C) or the anterior *gt* (B and D) domains.

These results are in apparent contradiction to a previous report indicating that the standard deviation of the location of the *hb* border was unchanged in single mutants for either *Kr* or *kni* [9]. We found the same result for single mutants (unpublished data) but in double mutants, uncharacterized previously, the standard deviation of the location of the anterior *hb* border doubles and that of the posterior border of the third anterior *gt* domain increases significantly (Figure 6). This experimental result strongly supports the model and rules out a picture in which Bcd provides the sole input to *hb* [16].

The same epistatic balance is seen in the posterior borders of the *Kr* and *kni* domains (Figure 4E, 4H, 4F, and 4I). Of the other correctly predicted domain borders, the anterior border of *kni* is set by repression from Kr and Hb (Figure 5A). Reduced levels of Bcd in the circuit cause this border to form at more anterior positions (Figure 5D); however, the shift is limited by increased levels of Kr and Hb repression toward the anterior (Figure 5E). Both borders of the posterior *gt* domain show epistatic balance (Figure 5F and 5G), but it does not correlate with position, because Bcd is not a morphogen in this region and does not provide positional information.

## Discussion

We have shown that the reduction in the variation of gene expression in the gap gene system takes place because of cross regulation between zygotic gap genes. Cross regulation is an intrinsic property of the system, and hence we have demonstrated that the system canalizes. More specifically, the experimentally supported analysis of the Results section “Variance Reduction by Gap Gene Cross Regulation and Experimental Verification” shows that the Bcd gradient is not sufficient to generate gap gene borders with the observed low variation.

Our results, together with those of other investigators, rule out three classes of theoretical models that have been invoked to settle certain questions associated with the positional variation of *hb*. The first two classes of models invoke either active transport [13] or a second gradient at the posterior pole of the embryo [12,14,15] as a mechanism for the reduction in variation of the *hb* border. With one exception [15], these studies only considered the *hb* border, while the work reported here correctly predicts the variation of six borders, some of which are not under the control of *bcd*. Moreover, the border positions in the model reported here scale with egg length (Figure 3E) without the need to invoke an undiscovered gradient. A third type of model [21] invokes transient behavior of the Bcd gradient and its associated thresholds during cycles 10–13 as a mechanism of variance reduction. This model has been invalidated by the experimental demonstration that intranuclear Bcd concentrations are in fact constant during this period of time [44]. Furthermore, threshold concentrations of Bcd propagate toward the posterior of the embryo during the establishment of the Bicoid gradient, but gap domains in the posterior, in fact, shift in an anterior direction [35]. A third contradiction with experiment is that the model used by the authors of [21] produces incorrect mutually exclusive gap domains in the presence of diffusion.

Many seemingly puzzling experimental results concerning the variance of expression borders driven by Bcd can be understood in a simple and unified manner by considering our results together with two recent studies of the Bcd gradient, one in vivo [16] and the other in fixed tissue [18]. Our comparison of Bcd-GFP data with fixed tissue *hb* data (Figure 1) shows that the range of *x*_Bcd_ in vivo is 10% EL, but that of *x*_Hb_ in fixed tissue data is 5% EL. This comparison finds support in the fixed-tissue study [18] in which a modified staining protocol was used and data were acquired in a single imaging cycle to minimize experimental error. The Bcd data thus obtained had the same level of error as Bcd-GFP data [16], yet a direct comparison between the Bcd and Hb data showed that the posterior border of the anterior *hb* domain was half as variable. The observation that the range of *x*_Bcd_ is twice that of *x*_Hb_ is extremely important, because there are many experimental manipulations that can double the range or standard deviation of *x*_Hb_, making it equivalent to the range or standard deviation of *x*_Bcd_.

First, these results indicate that the increased variance of *x*_Hb_ in *Kr;kni* double mutants (Figure 6A caption) can be interpreted as arising from the unmodified action of Bcd without reduction in variance mediated by other genes. This is a key point, because the larger standard deviation of *x*_Bcd_ observed in data from fixed tissue here and elsewhere [9] appeared to imply that variance reduction still took place in double mutants, albeit at a reduced level. Second, a 9% range of border positions has been observed from reporters driven by a fragment of the *hb* promoter believed to contain only Bcd binding sites (see Figure 4 in [21]), suggesting that this expression reflects the underlying variation of *x*_Bcd_. Third, when chromosome arm 3L is completely removed [9], the standard deviation of *x*_Hb_ doubles. We suggest that all three of these experimental manipulations uncover unmodified action of Bcd on *hb* by removing additional modulators respectively in *trans*, in *cis*, and by a currently uncharacterized mechanism.

Other important experimental results can be understood by recalling that the maximum accuracy of the Bcd gradient is inversely proportional to the square root of the Bcd concentration [16,19,60], suggesting that borders under unmodified Bcd control will have lower variation at more anterior positions. This fact explains the observation that a synthetic reporter construct driven only by Bcd has the same standard deviation as *x*_Hb_ [59]. The synthetic construct forms its border at 28.6% EL, where the in vivo data (see Figure 2B in [16]) indicate that the Bcd concentration is about three times greater than at the native *hb* border. If the standard deviation of 2.2% EL seen here in *Kr;kni* double mutants is the unmodified response to Bcd at 47% EL, then the expected standard deviation at 29% EL is 1.3%, which is close to the 1.6% observed (see Figure 5C in [59]). Finally, the fact that the standard deviation of the posterior border of the anterior *gt* domain increases by less than that of *hb* in *Kr;kni* mutant embryos can be understood by noting that it is located at about 40% EL. At this position, the in vivo data appear to indicate that the Bcd concentration is 1.69 times greater than that at the *hb* border. This concentration ratio predicts that the standard deviation of this border in *Kr;kni* double mutants will be 1.7% EL, while the actual value is 1.9% EL.

In recently published work, He et al*.* [18] report that the Bcd threshold at which the posterior border of the anterior *hb* domain forms is not constant but varies in a correlated manner with the slope of the Bcd profile. Figure 4D shows that in our simulations, the Bcd level at which the *hb* border forms changes as the Bcd profile is varied. Figure 4G shows that this effect is a consequence of the dynamic adjustment of the repression levels, arising from gap gene cross regulation. He et al. speculate that the stability of the Bcd protein might affect its ability to activate *hb*. However, our results suggest that the dynamic adjustment of the activation level is in fact an indirect consequence of gap gene cross regulation.

One remaining question concerns the reason for the correct prediction of gap gene domain border variation presented here, as it is clear that the fixed tissue Bcd data used for the theoretical study exaggerate the standard deviation of *x*_Bcd_ [16]. We believe that the high degree of canalization observed (Figure 3A) makes the theoretical model quite insensitive to initial variation, as is true of the biological system itself. In a companion work [61], we show that robust insensitivity to initial conditions is an inherent mathematical property of the gene circuit equations for this system. Moreover, the observed in vivo standard deviation of *x*_Bcd_ was obtained in an approximately isogenic stock grown at fixed temperature, while natural populations have much more variable genotypes and develop in the presence of temperature fluctuations. These factors alter the profile of the Bcd gradient but have little effect on the border position of downstream targets [9,62]. We believe that the fixed-tissue Bcd profiles used in the present study capture this extra level of variability characteristic of natural populations.

This analysis of canalization has focused on the reduction of variation of border positions of the gap genes with respect to the threshold positions of the maternal determinant Bcd (Results sections “Simulation of Bcd Variation and Size Variation” and “Variance Reduction by Gap Gene Cross Regulation and Experimental Verification”). The gap gene borders are specified at a low enough molecular number of Bcd [16] that intrinsic fluctuations are a significant source of variation [16,60]. The buffering of phenotypic variation against intrinsic fluctuations in molecular number, while not considered by Waddington, is likely to be an important aspect of canalization in biological systems. It is noteworthy that we were able to model canalization using a deterministic model with what were, essentially, stochastic initial conditions. Future work will need to treat the role of intrinsic fluctuations in the formation of maternal gradient systems [63,64]. While stochastic interactions undoubtedly occur among zygotic gap gene products, understanding the functional role of stochastic processes in the segmentation system will require the formulation of a fully stochastic model. Such a model must also produce the observed reduction in variation in the gap gene system, although the numerical details will differ.

Waddington hypothesized that canalization arises from gene interactions [1,65–68]. We have identified specific regulatory interactions among the gap genes responsible for canalization. It is noteworthy that variation reduction is an emergent property of the system and does not require special built-in mechanisms such as a posterior gradient. The mutual repression between the gap genes that is required for correct spatiotemporal expression is also responsible for canalization, providing support for the “intrinsic” [67] or “single-mode” [69] canalization hypothesis.

Our results make it possible to characterize in a more precise way how canalization works. The reduction of gap gene expression variation over time [6] suggests that canalization occurs because the possible developmental trajectories of the system tend to converge over time, thus reducing variation. In an accompanying manuscript [61], we show that the trajectories of the gap gene system in individual nuclei do tend to converge and are stable against perturbations. This convergence and stability are a consequence of the fact that the regulatory interactions of the gene circuit cause the state of the system to approach a stable steady state [70,71], also called an attractor. A given nucleus can potentially approach one of many attractors, a property called multistability, and a particular attractor is chosen based on the initial state of the nucleus. Waddington inferred canalization, in part, from the observation that there are discrete tissue types rather than a continuous blend. Multistability provides a rigorous mathematical justification for the connection between canalization and the discreteness of tissue type. If the initial state is perturbed by a small amount, the system is attracted by the same attractor, providing variation reduction. However, if the perturbation is large enough, or if attractors are created or destroyed, the trajectory can switch to a different attractor providing a discrete response. Our analysis [61] suggests that switching between attractors occurs in the gap gene system and is the mechanism by which the posterior border of the anterior *hb* domain forms. These results show that the gap gene network is a system with the intrinsic property of reducing variation, a form of error correction, and that this canalization property is a consequence of the mutual regulatory actions of the gap gene network.

## Methods

### Experimental methods.

The methods used to obtain and characterize the quantitative data are as described in earlier work [6]. All gene expression levels are on a scale of 0–255, chosen to maximize dynamic range without saturation. The *Kr*;*kni* double mutant embryos were made by crossing *Kr* ^1^ and *Df(3L)ri-79c* flies. 3–4-hours-old embryos were fixed and stained as described [72] using guinea pig anti-Kr and anti-Kni antibodies with Alexa Fluor 488 conjugated secondary antibody, rabbit anti-Gt and rat anti-Hb antibodies with Alexa Fluor 555 and 647 conjugated secondary antibodies (Molecular Probes), respectively. Embryos null for Kr and Kni showed no signal on the first channel. Double mutants in lateral orientation were scanned using a Leica TCS SP2 confocal microscope and the images were then processed as described [73]. Domain border positions for wild type and mutants were determined by calculating the A–P position at which the relative concentration is half its maximum value in the domain. The local maximum in a domain was determined using a quadratic spline approximation [74].

### Numerical methods.

The numerical implementation of the gene circuit equations is as described [35,36] with the addition of time varying external inputs (see Section S1.2 in Protocol S1). The gap gene circuit was fit to integrated gap gene data [6] using Parallel Lam Simulated Annealing (PLSA) [28,29]. PLSA minimizes the root mean squared (RMS) difference between model output and data. The minimized RMS difference for a circuit is a measure of the quality of fit, and is called the RMS score. The RMS score indicates the average difference between the model and the data. Search spaces, penalty function, and other annealing parameters were as described [27,36]. The circuit analyzed in detail had an RMS score of 10.76, corresponding to a proportional error in expression residuals of about 4–5%. Protocol S1 (Section S1.3) contains further details.

## Supporting Information

Protocol S1 Detailed Description of Gene Circuits, Bcd Variation Simulations, and Regulatory Analysis (148 KB PDF)

## Abbreviations

A–P: anteroposterior
*bcd*: *bicoid*
*cad,*: *caudal*
EL: egg length
GFP: green fluorescent protein
*gt*: *giant*
*hb*: *hunchback*
*kni*: *knirps*
*Kr*: *Krüppel*
*tll*: *tailless*
