# Supplementary material for: Canalization of Gene Expression in the Drosophila Blastoderm by Gap Gene Cross Regulation
Source: PLoS Biol. 2009 Mar 10;7(3):e1000049. doi: 10.1371/journal.pbio.1000049 (PMC2653557; doi:10.1371/journal.pbio.1000049)
Supplement: Supplementary file 1 [file pbio.1000049.sd001.pdf]

Canalization of Gene Expression in the *Drosophila* Blastoderm by Gap Gene Cross Regulation

Supplementary Information

Manu[1,7], Svetlana Surkova[2,7], Alexander V. Spirov[1], Vitaly V. Gursky[3], Hilde Janssens[6], Ah-Ram Kim[1], Ovidiu Radulescu[4], Carlos E. Vanario-Alonso[1], David H. Sharp[5], Maria Samsonova[2] & John Reinitz[1*]

[1]Department of Applied Mathematics and Statistics, and Center for Developmental Genetics, Stony Brook University, Stony Brook, NY 11794-3600, U.S.A.

[2]Department of Computational Biology, Center for Advanced Studies, St. Petersburg State Polytechnical University, St. Petersburg, 194064 Russia

[3]Theoretical Department, The Ioffe Physico-Technical Institute of the Russian Academy of Sciences, 26 Politekhnicheskaya St., St. Petersburg, 194021 Russia

[4]Institute of Mathematical Research of Rennes, University of Rennes 1, 35042 Rennes Cedex, France

[5]Theoretical Division, Los Alamos National Laboratory, Los Alamos, NM 87545, U.S.A.

[6]EMBL/CRG Research Unit in Systems Biology CRG – Centre de Regulació Genòmica, Room 571 Dr. Aiguader 88, 08003 Barcelona, Spain

[7]These authors contributed equally to this paper.

*To whom correspondence should be addressed. E-mail: reinitz@odd.bio.sunysb.edu

Figure, table and section numbers in this text are prefixed with the letter "S". Figures, tables and sections from the main text are referenced without an alphabetical prefix.

Table of Contents

S1 Gap gene circuits **5**

S1.1 Equations 6

S1.2 Numerical implementation of time varying external inputs 7

S1.3 Optimization and selection procedure for gap gene circuits 10

S2 The simulation of Bcd variation **14**

S3 Regulatory analysis of gap gene borders **14**

List of Figures

S1 The regulation-expression function $g(u)$ 6
S2 Division schedule and time classes 8
S3 Interpolation of time varying external inputs from data 9
S4 Gap gene expression patterns in the gene circuit chosen for analysis 11

List of Tables

S1 Regulatory parameters of the gap gene circuit chosen for analysis 12

S2 Kinetic parameters of the gap gene circuit chosen for analysis 12

S3 Equation parameters for the 23 gap gene circuits that have consistent network topology 13

S4 Positional variation of gap gene borders in circuits produced with Bcd gradients other than the median 14

S1 Gap gene circuits

A gene circuit [1–5] determines the time evolution of protein concentrations in the syncytial blastoderm of *Drosophila melanogaster*. The circuits used in this paper comprise the gap genes *hb*, *Kr*, *gt*, and *kni* of the anteroposterior segmentation system. Their protein products are transcription factors that localize in the nuclei of the blastoderm [6–9]. Therefore, the state variables are the concentrations of the protein products of these genes inside nuclei. Anteroposterior (A–P) and dorsoventral (D–V) patterning systems are largely independent of each other in the presumptive germ band of the blastoderm embryo. This allows us to consider a one-dimensional row of nuclei along the anteroposterior axis of the embryo, from 35% EL to 92% EL. The modeled region extends over 58% of the A–P axis, from the peak of the third *gt* stripe to the posterior border of the posterior *hb* domain (Fig. S4B).

The circuit functions according to three rules: interphase, mitosis and division. The first two rules describe the continuous dynamics of proteins during interphase and mitosis. During interphase the evolution of protein concentrations is determined by three processes: regulated protein synthesis, protein transport, and protein decay. During mitosis, transcription shuts down and nascent transcripts are destroyed [10]. Therefore, only protein transport and protein decay govern the dynamics in the mitosis rule.

The third rule, division, accounts for nuclear cleavages in the blastoderm. It models mitotic division as a discontinuous change in the state of the system. At the end of a mitosis, each nucleus is replaced with its daughter nuclei. The inter-nuclear distance is halved and the daughter nuclei inherit the state of the mother nucleus. The divisions are carried out according to a division schedule based on experimental data [11] (Fig. S2).

The gap gene circuits used in this study model events occurring during a period of time which begins at the onset of cleavage cycle 13 and ends at the onset of gastrulation at the end of cycle 14A [11]. *Kr*, *gt*, and *kni* are exclusively zygotic, and their expression at the protein level is first detectable at early cycle 13 [12–17]. *hb*, which is expressed both maternally and zygotically, shows a large increase in expression in cycle 13 [18], indicating commencement of its zygotic expression. Time t is measured in minutes from the start of cleavage cycle 13. The interphase of cycle 13 lasts for 16.0 min, and its mitosis from 16.0 to 21.1 min. At $t = 21.1$ min, the thirteenth division is carried out by applying the division rule. The interphase of cycle 14A starts immediately after division, and lasts until gastrulation at $t = 71.1$ min.

S1.1 Equations

The two continuous rules, interphase and mitosis, use a system of ordinary differential equations (ODEs) to describe the dynamics of protein concentrations. Let there be M nuclei in the modeled region during a particular cleavage cycle. Let i denote a particular nucleus, counting from anterior to posterior. We denote a particular segmentation gene by $a \in 1, \ldots, N$, where N genes are represented in the circuit. $v_i^a(t)$ is the protein concentration of gene a in nucleus i. The time evolution of the state variables $v_i^a(t)$ is given by the solution of the system of $M \times N$ ODEs,

$$\frac{dv_i^a}{dt} = R^a g\left(\sum_{b=1}^{N} T^{ab} v_i^b + m^a v_i^{\mathrm{Bcd}} + \sum_{\beta=1}^{N_e} E^{a\beta} v_i^\beta(t) + h^a\right) + D^a(n)\left[(v_{i-1}^a - v_i^a) + (v_{i+1}^a - v_i^a)\right] - \lambda^a v_i^a. \quad \text{(S1)}$$

The first term on the right hand side of Eq. (S1) represents protein synthesis, the second one protein transport through Fickian diffusion and the last term represents first-order protein degradation.

The protein synthesis rate for gene a is determined by the maximum synthesis rate R^a and the regulatory input to a, $u^a \equiv \sum_{b=1}^{N} T^{ab} v_i^b + m^a v_i^{\mathrm{Bcd}} + \sum_{\beta=1}^{N_e} E^{a\beta} v_i^\beta(t) + h^a$. The rate of protein synthesis is the product of R^a and the regulation-expression function $g(u^a) = \frac{1}{2}\left[\left(u^a/\sqrt{(u^a)^2+1}\right)+1\right]$ (see Fig. S1).

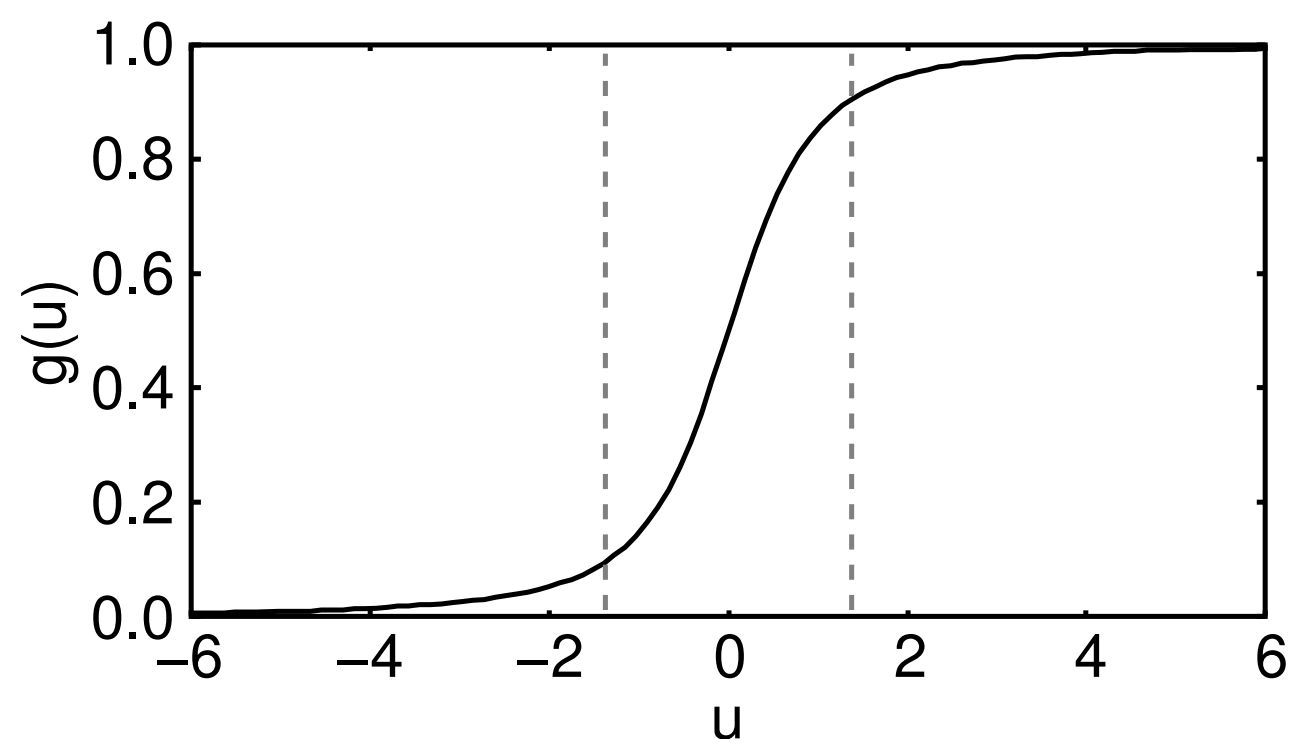

Figure S1: The regulation-expression function $g(u)$. The dashed vertical lines are at values of total input u at which synthesis rate is 10% or 90% of maximum.

The regulatory input u^a in turn accounts for the transcriptional regulation of gene a by transcription factors. Each term of u^a corresponds to a distinct type of factor. The first term $\sum_{b=1}^{N} T^{ab} v_i^b$ represents the regulation of gene a by the genes $b \in 1, \ldots, N$ of the circuit. The elements of the ge-

netic interconnection matrix T, T^{ab}, characterize the regulatory effect of protein b on the synthesis of gene a. A positive value of T^{ab} represents activation and a negative value represents repression.

Spatially and temporally homogeneous maternal factors are represented in u^a via its fourth term, h^a. The second term of u^a represents the action of Bcd, which has a concentration that is spatially inhomogeneous, but constant in time [16]. m^a is the strength of the regulation of gene a by Bcd, while v_i^{Bcd} is the concentration of Bcd in nucleus i.

Finally, we represent the effects of transcription factors which vary over space and time and regulate gap gene expression but are not themselves regulated by the gap genes through the third term of u^a, $\sum_{\beta=1}^{N_e} E^{a\beta} v_i^{\beta}(t)$. N_e is the number of such factors in the circuit, $E^{a\beta}$ is the regulatory effect of the time varying external input β on gene a, and $v_i^{\beta}(t)$ is the concentration of external input β in nucleus i at time t. The gene circuit used in this study has two such factors, Cad ($v_i^{\mathrm{Cad}}(t)$) and Tll ($v_i^{\mathrm{Tll}}(t)$). *cad* is expressed from both the maternal and zygotic genomes. However, its expression pattern is not affected in mutants for *hb*, *Kr*, *gt* and *kni*, except for a slight expansion of the posterior stripe in *hb*$^-$ during late cycle 14 [19]. Similarly, *tll* expression is not affected in these mutants [20]. The concentrations of time varying external inputs Cad and Tll were determined by interpolation from data [16] (see Section S1.2 for details).

The second term of Eq. (S1) represents protein transport between nuclei as spatially discretized Fickian diffusion. The diffusion parameter D^a is assumed to vary inversely with the squared distance between neighboring nuclei. The third term is protein degradation, in which λ^a is the decay rate of the product of gene a. It is related to the protein half life of the product of gene a by $t_{1/2}^a = \ln 2/\lambda^a$.

Since Kr, Gt, and Kni proteins first appear only in cycle 13, they have initial conditions of zero, that is $v_i^{Kr}(0) = v_i^{gt}(0) = v_i^{kni}(0) = 0$. For *hb*, the expression data from cycle 12 is used as the initial condition. These data are the maternal component of *hb* since its expression intensifies only in cycle 13.

S1.2 Numerical implementation of time varying external inputs

In order to specify the right hand side of Eq. (S1) fully, the concentrations of the time varying external inputs, Cad ($v_i^{\mathrm{Cad}}(t)$) and Tll ($v_i^{\mathrm{Tll}}(t)$), must be supplied for any time in the duration of the model. Average concentrations for these genes are known at ten time points $t_k, k = -1, 0, \ldots, 8$. $t_{-1} = 0$ min, t_0 is the midpoint of cycle 13, and $t_1, \ldots, t_8$ correspond to the eight time classes T1–T8 in cycle 14 (Table 1). For nucleus i and external input β, let $v_i^{\beta}(t_k) = v_{i,k}^{\beta}$, where $k = -1, 0, \ldots, 8$. The concentration of external input β in nucleus i is then determined at an arbitrary time t by piecewise linear interpolation,

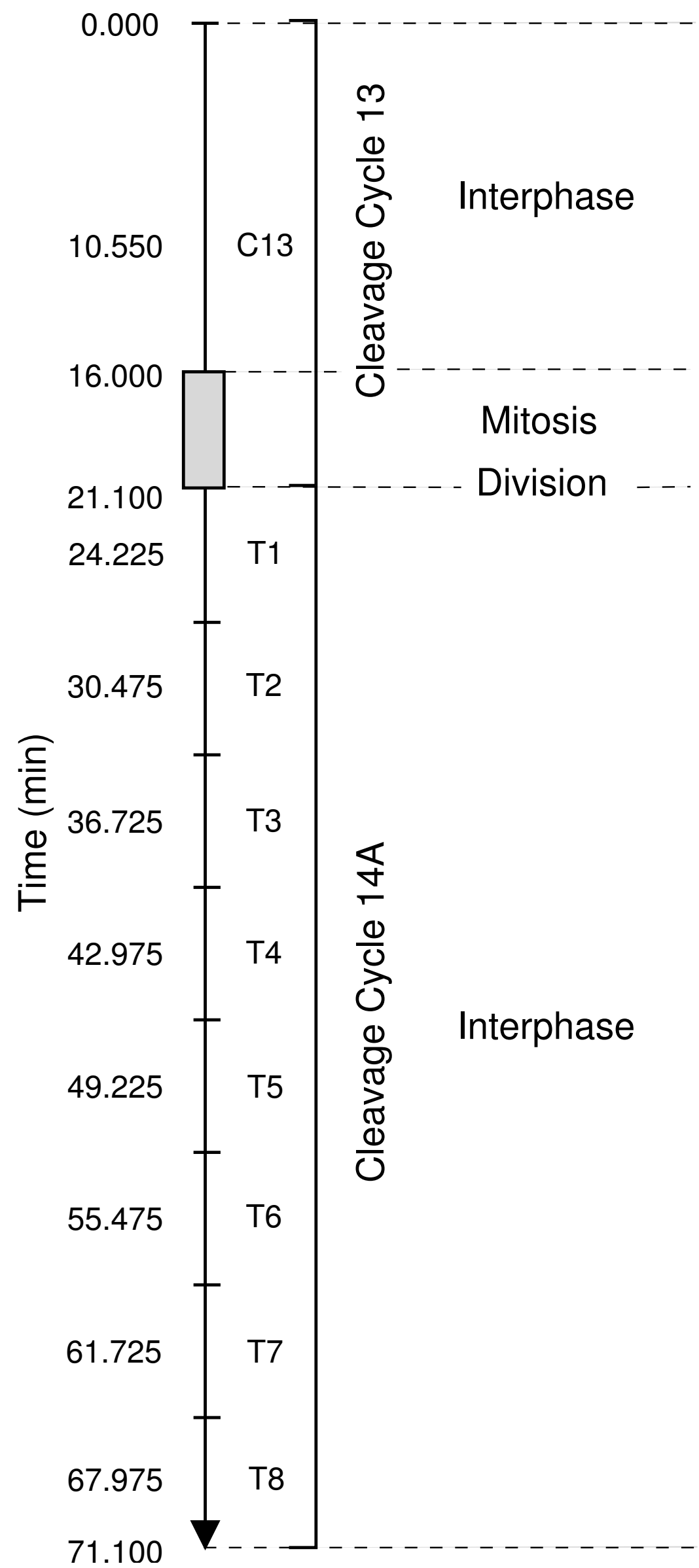

Figure S2: Division schedule and time classes. $t = 0$ is at the start of cleavage cycle 13. The model runs until gastrulation at $t = 71.1$ min. The duration of the cleavage cycles and mitosis was determined from experimental data [11]. The dashed lines demarcate time intervals in which different rules of the model apply. The intervals are labeled with the rules on the right. The nine time points at which model is compared to data are indicated. There is one time point for cycle 13 (C13), and eight points for cycle 14A (time classes T1–T8). Figure reproduced here with the permission of J. Jaeger [4].

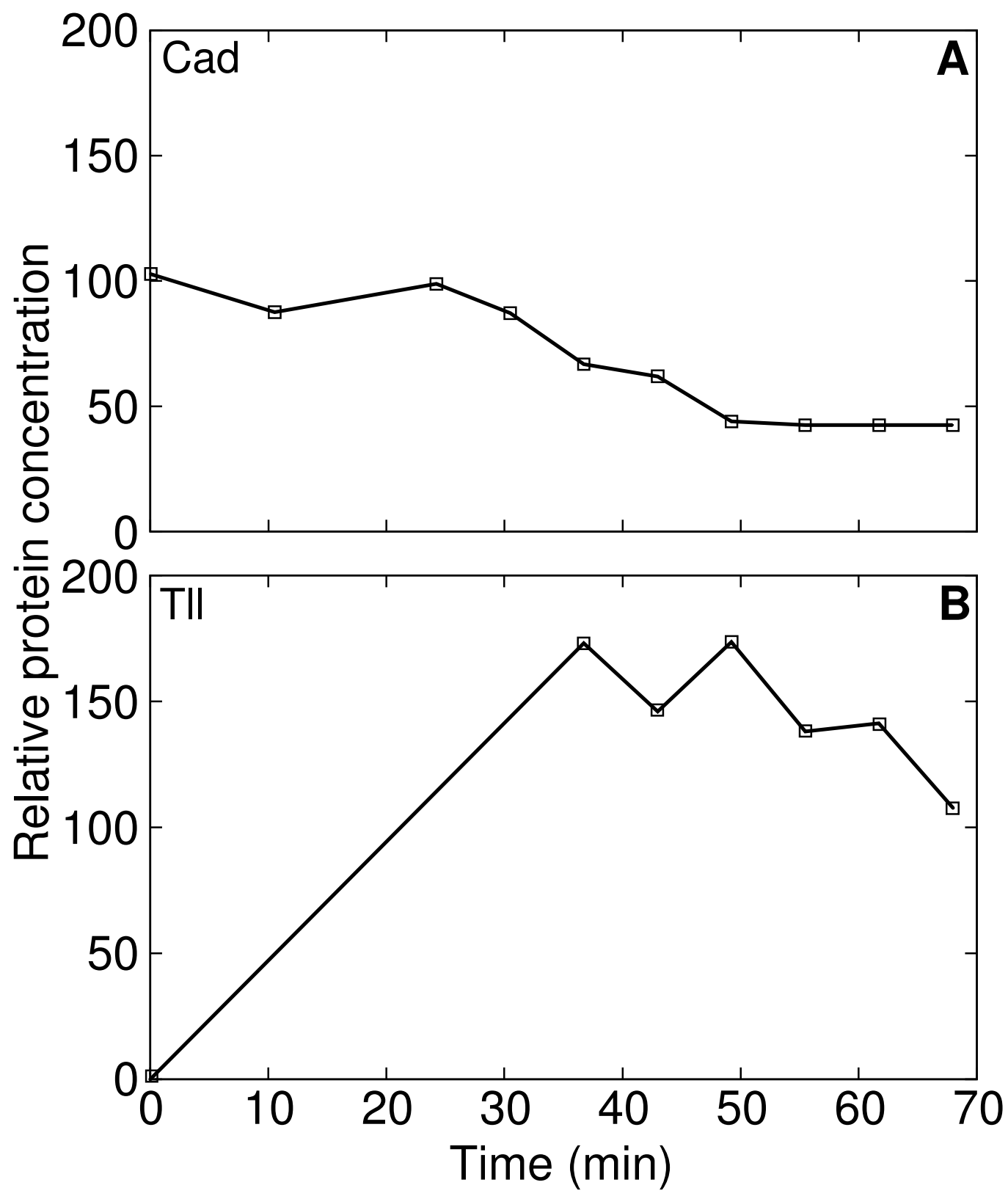

Figure S3: Interpolation of time varying external inputs from data. (A) Cad data and linearly interpolated time profile at 50% EL. (B) Tll data and linearly interpolated time profile at 92% EL. Square boxes indicate data points. Cad concentration at $t = 0$ min is determined from cleavage cycle 12 data. Tll concentration is zero at 0 min since Tll is first detected in cleavage cycle 13 [16]. The other time points are midpoints of time classes (Table 1). Tll data were only available for time classes T3–T8. Lines are interpolated concentrations.

$$v_i^\beta(t) = \frac{(t_{k+1} - t)v_{i,k}^\beta + (t - t_k)v_{i,k+1}^\beta}{t_{k+1} - t_k}, \quad t_k \le t \le t_{k+1}.$$

Fig. S3 shows such interpolation at 50% EL for Cad, and at 92% EL for Tll. Higher order methods like cubic splines were not used because they gave rise to artifacts from experimental noise.

S1.3 Optimization and selection procedure for gap gene circuits

The Parallel Lam Simulated Annealing algorithm [21, 22] produces many candidate circuits, and we selected a circuit using a three-step method [5]. First, only circuits with an RMS score less than 12.0 were considered. These circuits were screened further for patterning defects, and any circuit with major defects was discarded. Finally, experimental [23] and theoretical [24] investigations have shown that *Kr* represses *hb*, therefore, we only select circuits for further consideration if they show this property.

With this screening process, we obtained 23 circuits out of a total of 65 optimizations. This set of circuits have the same network topology (Table S3) as the circuits studied in earlier work [5, 24]. The signs of the regulatory parameters are the same in all circuits with three exceptions, $T^{gt \leftarrow kni}$, $T^{Kr \leftarrow gt}$, and m^{kni}. Of these three, the last two change sign in only one circuit, `k1_014` (Table S3). This level of consistency suggests that network topology has been inferred at a qualitative level, in agreement with the conclusions of a recent study of parameter determinability of gap gene circuits [25]. Bcd and Cad are activators of *hb*, *Kr*, *gt*, and *kni*. *tll* is an activator of *hb*, and a repressor of the other gap genes. The interaction between *hb*, *Kr*, *gt*, and *kni* is one of mutual repression, with two exceptions: (1) *gt* is an activator of *hb* in all the circuits obtained. (2) *kni* is an activator of *gt* in about half the circuits, and a repressor in the other half. This network topology is discussed in depth elsewhere [5, 24]. These results also hold true for circuits obtained using other Bcd profiles from the middle of the parameter scatter in Fig. 2A (black circles).

We chose one circuit (`k1_007`), which has RMS score 10.76, for further analysis. Its parameters are shown in Tables S1 and S2. The chosen circuit's gap gene patterns (Fig. S4A,B) are consistent with data except for two minor defects. The first one is a bulge on the anterior border of the posterior *hb* domain. The second is that the posterior border of the posterior *hb* domain does not form fully. Some circuits reported in previous work [5] also suffer from these defects. The first defect is due to very low levels of spurious *tll* expression in the middle part of the embryo stemming from imperfect background removal. The second is due to the omission of the gene *hkb* [20, 26, 27] from the model. *hkb* is a terminal gap gene expressed near the posterior pole.

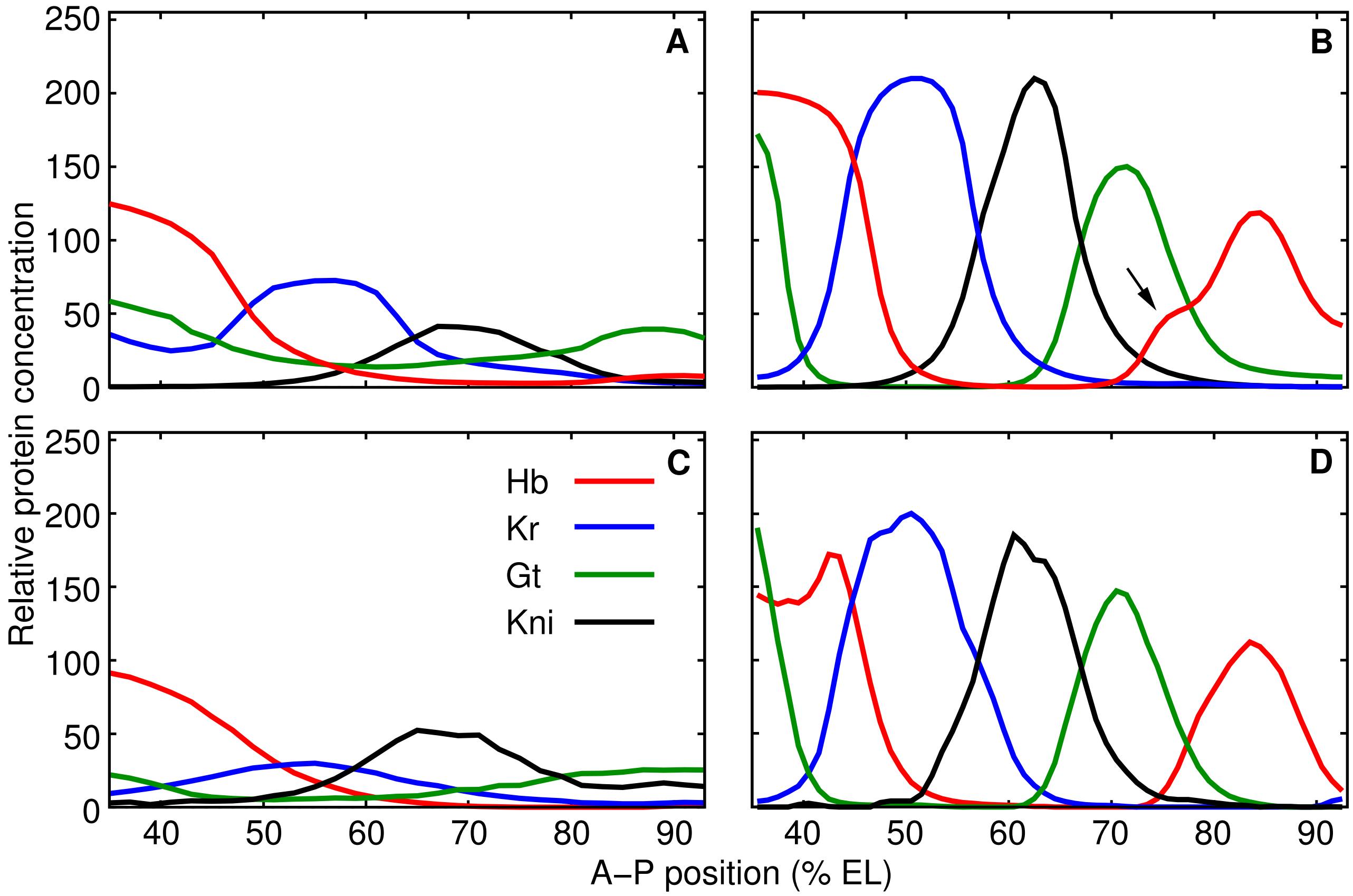

Figure S4: Gap gene expression patterns in the gene circuit chosen for analysis. Cleavage cycle 13 (A) and time class T8 (B) model output. The arrow shows the main patterning defect. (C,D) Averaged gene expression data for cleavage cycle C13 (C) and time class T8 (D).

Target gene a	**Regulator gene** b *bcd*	*cad*	*tll*	*hb*	*Kr*	*gt*	*kni*
hb	0.025	0.004	0.003	0.021	–0.001	0.022	–0.112
Kr	0.118	0.021	–0.203	–0.026	0.035	–0.042	–0.062
gt	0.256	0.023	–0.011	–0.028	–0.202	0.007	0.003
kni	0.012	0.020	–0.187	–0.082	0.000	–0.017	0.013

Table S1: Regulatory parameters of the gap gene circuit chosen for analysis. The second, third, and fourth columns show m^a, $E^{a \leftarrow cad}$, and $E^{a \leftarrow tll}$ respectively. The columns of the 4×4 matrix T^{ab} are shown in the last four columns of the table. The parameters are explained in Section S1.

**Parameter**	**Gene** a *hb*	*Kr*	*gt*	*kni*
R^a	15.000	10.354	15.000	15.000
D^a	0.166	0.200	0.103	0.200
$t^a_{1/2}$	9.529	15.908	9.438	13.062

Table S2: Kinetic parameters of the gap gene circuit chosen for analysis. h^a was kept fixed at -2.5 during optimization. The parameters are explained in Section S1.

parameter	007	011	013	016	022	026	029	031	042	045	053	080	082	083	084	095	101	014	027	051	065	071	079
R^{hb}	15.000	15.000	15.000	15.000	15.000	15.000	15.000	15.000	15.000	15.000	15.000	15.000	15.000	15.000	15.000	15.000	15.000	15.000	15.000	15.000	15.000	15.000	15.000
R^{Kr}	10.354	10.374	13.478	10.359	11.550	10.475	11.321	12.950	15.000	15.000	10.956	11.661	10.437	12.095	10.326	13.080	15.000	10.000	11.445	14.974	15.000	15.000	14.513
R^{gt}	15.000	15.000	14.848	15.000	15.000	15.000	15.000	15.000	15.000	15.000	15.000	14.970	15.000	15.000	15.000	14.411	13.471	13.274	15.000	13.979	15.000	15.000	14.344
R^{kni}	15.000	15.000	15.000	15.000	15.000	15.000	15.000	15.000	15.000	11.751	15.000	15.000	15.000	13.131	15.000	15.000	15.000	10.000	13.090	15.000	15.000	15.000	15.000
$T^{hb \leftarrow hb}$	0.021	0.021	0.020	0.021	0.021	0.021	0.021	0.021	0.019	0.020	0.021	0.021	0.021	0.021	0.021	0.021	0.019	0.019	0.021	0.020	0.019	0.019	0.020
$T^{hb \leftarrow Kr}$	-0.001	-0.001	-0.002	-0.001	-0.001	-0.001	-0.001	-0.002	-0.001	-0.001	-0.001	-0.001	-0.001	-0.002	-0.001	-0.002	-0.001	-0.001	-0.001	-0.002	-0.001	-0.001	-0.002
$T^{hb \leftarrow gt}$	0.022	0.022	0.016	0.022	0.019	0.021	0.020	0.018	0.019	0.019	0.021	0.019	0.021	0.015	0.022	0.019	0.017	0.022	0.015	0.017	0.017	0.017	0.017
$T^{hb \leftarrow kni}$	-0.112	-0.111	-0.085	-0.111	-0.097	-0.110	-0.105	-0.097	-0.130	-0.125	-0.109	-0.095	-0.110	-0.115	-0.113	-0.099	-0.110	-0.147	-0.117	-0.084	-0.103	-0.099	-0.084
$T^{Kr \leftarrow hb}$	-0.026	-0.026	-0.016	-0.026	-0.021	-0.025	-0.023	-0.019	-0.021	-0.019	-0.025	-0.020	-0.025	-0.019	-0.026	-0.015	-0.020	-0.050	-0.021	-0.011	-0.014	-0.013	-0.012
$T^{Kr \leftarrow Kr}$	0.035	0.035	0.020	0.035	0.029	0.034	0.029	0.021	0.016	0.015	0.030	0.028	0.035	0.028	0.035	0.021	0.015	0.079	0.030	0.019	0.016	0.016	0.020
$T^{Kr \leftarrow gt}$	-0.042	-0.042	-0.054	-0.042	-0.045	-0.041	-0.053	-0.064	-0.108	-0.100	-0.057	-0.045	-0.041	-0.037	-0.042	-0.047	-0.124	0.010	-0.040	-0.044	-0.073	-0.074	-0.043
$T^{Kr \leftarrow kni}$	-0.062	-0.061	-0.032	-0.062	-0.048	-0.060	-0.048	-0.033	-0.025	-0.023	-0.051	-0.047	-0.061	-0.049	-0.062	-0.032	-0.025	-0.114	-0.053	-0.026	-0.024	-0.023	-0.028
$T^{gt \leftarrow hb}$	-0.028	-0.028	-0.036	-0.028	-0.032	-0.028	-0.030	-0.034	-0.026	-0.027	-0.029	-0.032	-0.028	-0.035	-0.028	-0.036	-0.034	-0.020	-0.033	-0.037	-0.030	-0.030	-0.036
$T^{gt \leftarrow Kr}$	-0.202	-0.202	-0.234	-0.202	-0.227	-0.205	-0.208	-0.218	-0.163	-0.178	-0.203	-0.228	-0.204	-0.243	-0.198	-0.228	-0.211	-0.240	-0.245	-0.246	-0.202	-0.222	-0.247
$T^{gt \leftarrow gt}$	0.007	0.007	0.004	0.007	0.006	0.007	0.006	0.004	0.005	0.005	0.007	0.006	0.007	0.007	0.007	0.004	0.003	0.027	0.007	0.002	0.003	0.002	0.002
$T^{gt \leftarrow kni}$	0.003	0.003	-0.001	0.003	0.001	0.003	0.001	-0.000	0.001	0.001	0.002	0.001	0.003	-0.001	0.003	-0.001	-0.001	-0.015	0.001	-0.003	-0.000	0.001	-0.003
$T^{kni \leftarrow hb}$	-0.082	-0.083	-0.098	-0.083	-0.092	-0.085	-0.086	-0.087	-0.073	-0.073	-0.080	-0.092	-0.084	-0.018	-0.082	-0.095	-0.075	-0.020	-0.019	-0.109	-0.111	-0.117	-0.109
$T^{kni \leftarrow Kr}$	-0.000	-0.000	-0.003	-0.000	-0.002	-0.000	-0.001	-0.003	-0.003	-0.009	-0.001	-0.002	-0.000	-0.021	-0.000	-0.003	-0.004	-0.024	-0.021	-0.004	-0.004	-0.004	-0.003
$T^{kni \leftarrow gt}$	-0.017	-0.017	-0.019	-0.017	-0.019	-0.017	-0.018	-0.018	-0.016	-0.037	-0.017	-0.018	-0.017	-0.110	-0.017	-0.018	-0.016	-0.189	-0.106	-0.020	-0.018	-0.018	-0.020
$T^{kni \leftarrow kni}$	0.013	0.013	0.011	0.013	0.012	0.013	0.012	0.012	0.013	0.017	0.013	0.012	0.013	0.046	0.013	0.012	0.013	0.035	0.047	0.010	0.010	0.010	0.010
$T^{hb \leftarrow cad}$	0.004	0.004	0.005	0.004	0.005	0.004	0.005	0.005	0.006	0.006	0.004	0.005	0.004	0.006	0.004	0.004	0.007	0.008	0.006	0.006	0.006	0.007	0.005
$T^{hb \leftarrow tll}$	0.003	0.003	0.003	0.003	0.003	0.003	0.003	0.003	0.002	0.002	0.002	0.003	0.003	0.011	0.002	0.003	0.002	0.012	0.011	0.003	0.002	0.002	0.003
$T^{Kr \leftarrow cad}$	0.021	0.021	0.021	0.021	0.021	0.021	0.021	0.022	0.021	0.022	0.022	0.021	0.021	0.021	0.021	0.021	0.023	0.021	0.022	0.021	0.022	0.022	0.021
$T^{Kr \leftarrow tll}$	-0.203	-0.202	-0.101	-0.203	-0.158	-0.197	-0.175	-0.122	-0.180	-0.164	-0.189	-0.151	-0.199	-0.140	-0.208	-0.084	-0.119	-0.103	-0.141	-0.058	-0.082	-0.067	-0.062
$T^{gt \leftarrow cad}$	0.023	0.023	0.030	0.023	0.026	0.024	0.025	0.028	0.022	0.024	0.024	0.027	0.024	0.027	0.023	0.031	0.027	0.029	0.027	0.035	0.028	0.030	0.034
$T^{gt \leftarrow tll}$	-0.011	-0.011	-0.017	-0.011	-0.014	-0.011	-0.012	-0.015	-0.011	-0.013	-0.011	-0.014	-0.011	-0.021	-0.011	-0.017	-0.013	-0.034	-0.020	-0.020	-0.015	-0.015	-0.020
$T^{kni \leftarrow cad}$	0.020	0.020	0.020	0.020	0.020	0.020	0.020	0.020	0.018	0.021	0.020	0.020	0.020	0.031	0.020	0.020	0.018	0.046	0.030	0.021	0.019	0.019	0.021
$T^{kni \leftarrow tll}$	-0.189	-0.189	-0.166	-0.189	-0.174	-0.188	-0.185	-0.174	-0.201	-0.193	-0.187	-0.174	-0.188	-0.007	-0.190	-0.164	-0.160	-0.010	-0.006	-0.154	-0.180	-0.172	-0.153
m^{hb}	0.025	0.025	0.029	0.025	0.027	0.026	0.026	0.029	0.033	0.030	0.026	0.027	0.026	0.032	0.025	0.028	0.033	0.028	0.032	0.028	0.029	0.028	0.028
m^{Kr}	0.118	0.117	0.108	0.118	0.109	0.115	0.123	0.126	0.157	0.147	0.133	0.109	0.116	0.099	0.119	0.103	0.164	0.099	0.106	0.087	0.117	0.112	0.089
m^{gt}	0.256	0.257	0.299	0.256	0.279	0.258	0.265	0.289	0.219	0.230	0.260	0.278	0.258	0.298	0.254	0.303	0.278	0.154	0.294	0.305	0.255	0.257	0.303
m^{kni}	0.012	0.013	0.055	0.012	0.031	0.014	0.026	0.050	0.043	0.067	0.019	0.032	0.014	0.005	0.012	0.050	0.060	-0.013	0.013	0.065	0.084	0.091	0.063
D^{hb}	0.166	0.166	0.143	0.166	0.156	0.165	0.161	0.158	0.174	0.177	0.165	0.157	0.165	0.166	0.166	0.170	0.148	0.197	0.173	0.136	0.131	0.121	0.140
D^{Kr}	0.200	0.200	0.200	0.200	0.200	0.200	0.200	0.200	0.200	0.200	0.200	0.200	0.200	0.200	0.200	0.200	0.200	0.200	0.200	0.200	0.200	0.200	0.200
D^{gt}	0.103	0.103	0.107	0.103	0.109	0.103	0.107	0.110	0.106	0.107	0.106	0.108	0.103	0.123	0.102	0.103	0.107	0.200	0.119	0.095	0.111	0.112	0.101
D^{kni}	0.200	0.200	0.200	0.200	0.200	0.200	0.200	0.200	0.200	0.200	0.200	0.200	0.200	0.200	0.200	0.200	0.200	0.200	0.200	0.200	0.200	0.200	0.200
$t_{1/2}^{hb}$	9.529	9.528	9.495	9.528	9.523	9.528	9.529	9.506	9.599	9.586	9.531	9.525	9.529	9.537	9.528	9.481	9.586	9.631	9.538	9.480	9.563	9.563	9.487
$t_{1/2}^{Kr}$	15.908	15.868	11.582	15.899	13.784	15.670	14.140	12.086	10.052	10.041	14.713	13.619	15.742	13.176	15.960	11.929	10.086	15.820	14.169	10.199	10.212	10.253	10.555
$t_{1/2}^{gt}$	9.438	9.437	9.294	9.437	9.484	9.435	9.458	9.222	9.406	9.317	9.470	9.495	9.436	9.356	9.430	9.467	10.656	11.305	9.262	9.903	9.420	9.418	9.673
$t_{1/2}^{kni}$	13.062	13.068	13.091	13.064	13.213	13.091	13.160	12.844	12.811	15.983	13.077	13.169	13.084	12.321	13.057	12.928	12.459	18.000	12.322	13.384	13.397	13.471	13.384

Table S3: Equation parameters for the 23 gap gene circuits that have consistent network topology. h^{hb}, h^{Kr}, h^{gt}, and h^{kni} were fixed to -2.5. The circuit analyzed in this study, `k1_007`, is shown in the first column. See text for parameter definitions.

S2 The simulation of Bcd variation

Boundary	k1_007	k12_004		k13_015		k14_015		k15_004	
	raw	fit	raw	fit	raw	fit	raw	fit	raw
hb posterior	1.45	1.27	1.42	0.75	0.91	0.94	1.07	0.98	1.16
Kr posterior	0.78	0.58	0.74	0.51	0.58	0.41	0.48	0.59	0.61
kni anterior	0.96	0.62	0.89	1.00	0.99	0.38	0.68	1.13	0.99
kni posterior	1.05	1.17	0.86	0.89	0.86	0.90	1.03	1.28	1.15
gt anterior	1.11	0.95	0.99	1.04	1.39	1.30	1.49	1.37	1.44
gt posterior	1.56	1.30	1.51	0.57	1.59	1.44	1.93	1.08	1.83

Table S4: Positional variation of gap gene borders in circuits produced with Bcd gradients other than the median. Numbers are standard deviations in % EL. The top row shows the circuits; `k1_007` is the circuit produced with the median Bcd profile (Fig. 2B) and analyzed in this study. The other circuits were fit using the Bcd profiles highlighted in Fig. 2A with black circles. The first column shows the six borders that have low positional variation in the simulations of Bcd variation. Bcd variation was simulated in each circuit with two families of Bcd profiles. The first family consisted of raw, background-removed Bcd profiles (even-numbered columns). The second family consisted of the exponential fits of the profiles of the first family made using Eq. (2) (odd-numbered columns). The positional variation of the borders in simulations of circuit `k1_007` using the second family of Bcd profiles is given in Table 2.

S3 Regulatory analysis of gap gene borders

This section describes the methodology of the regulatory analysis presented in Section 2.3. At a border of the expression domain of gene a, the regulation-expression function $g(u^a)$ (see Eq. S1) changes from a value close to zero to a value close to one. In the regulatory analysis we determine the set of regulators of the gene a that are responsible for this change in the value of $g(u^a)$. Here, $u^a = \sum_{b=1}^{N} T^{ab} v_i^b + m^a v_i^{\mathrm{Bcd}} + \sum_{\beta=1}^{N_e} E^{a\beta} v_i^{\beta}(t) + h^a$ is the total regulatory input to the gene a. Let i_B be the indices of the nuclei over which the border of gene a forms. Since the sigmoid $g(u^a)$ is approximately linear between 10% and 90% expression levels (Fig. S1), we can write $g(u^a_{i_B}) \approx u^a_{i_B} = \sum_{b=1}^{N} T^{ab} v^b_{i_B} + m^a v^{\mathrm{Bcd}}_{i_B} + \sum_{\beta=1}^{N_e} E^{a\beta} v^{\beta}_{i_B} + h^a$ for the purposes of this analysis.

Consider the posterior border of the expression domain of a gap gene a. Let the expression level of protein a be at 90% of maximum at $i = i_1$, and at 10% maximum at $i = i_M$. The analysis is similar for anterior borders, but with $i = i_1$ at 10% maximum, and $i = i_M$ at 90% maximum. The total change in u^a is $u^a_{i_1} - u^a_{i_M}$. Since this change is just a sum of regulatory contributions, we can divide it into individual regulatory terms

$$u^a_{i_1} - u^a_{i_M} = \sum_{b=1}^{N} T^{ab}(v^b_{i_1} - v^b_{i_M}) + m^a(v^{\text{Bcd}}_{i_1} - v^{\text{Bcd}}_{i_M}) + \sum_{\beta=1}^{N_e} E^{a\beta}(v^\beta_{i_1} - v^\beta_{i_M}). \tag{S2}$$

By comparing the magnitude of the change in different regulatory terms ($T^{ab}(v^b_{i_1} - v^b_{i_M})$, $m^a(v^{\text{Bcd}}_{i_1} - v^{\text{Bcd}}_{i_M})$, $E^{a\beta}(v^\beta_{i_1} - v^\beta_{i_M})$), we can determine which regulators are driving the formation of the boundary. In Fig. 4A–C and 5A–C, the spatial derivatives of u^a and individual regulatory terms are plotted so that the total change in u^a is the area below or above the curve, and the total change in each regulatory term is the area between curves.

It is possible to simplify this analysis by eliminating many regulators that cannot set the border. Autoregulation, for instance, cannot form the border [5]. It can only make a border sharper. Since this is a posterior border, the concentration of a reduces as one goes from anterior (i_1) to posterior (i_M). An activator whose concentration is increasing from i_1 to i_M will tend to counteract the reduction in a's concentration. Thus an activator whose gradient falls in the opposite direction of the border cannot aid its formation. Similarly a repressor whose concentration is decreasing from i_1 to i_M, that is, a repressor gradient in the same direction as the border, cannot cause the border to form.

Such simplification is also possible for anterior borders (increasing from i_1 to i_M). Activator gradients in the opposite direction to the border, and repressor gradients in the same direction as the anterior border cannot cause it to form.

Here we list the regulatory inputs that were eliminated in the regulatory analysis shown in Fig. 4A–C and Fig. 5A–C following the procedure described above. For the posterior border of the anterior *hb* domain (Fig. 4A), Hb autoactivation and Cad activation were eliminated. Of these two, Cad activation has a negligible contribution and hence only Hb activation is shown in red. In the analysis of the regulation of the posterior border of the central *Kr* domain (Fig. 4B), Kr autoactivation, Cad activation, Tll repression, and Hb repression were eliminated and their combined contribution is shown in red. For the posterior border of the posterior *kni* domain (Fig. 4C), Kni autoactivation, Kr repression, and Cad activation cannot set the border and their contribution is shown in red.

In the analysis of the regulation of the anterior border of the posterior *kni* domain (Fig. 5A), Kni autoactivation, Bcd activation, and Tll repression were eliminated and are shown in red. For the anterior border of the posterior *gt* domain (Fig. 5B), Gt autoactivation, Bcd activation, Kni activation, and Tll repression were eliminated and are shown in red. For the posterior border of the posterior *gt* domain (Fig. 5C), Gt autoactivation, Cad activation, and Kr repression cannot set the border and are shown in red.

References

[1] Mjolsness E, Sharp DH, Reinitz J (1991) A connectionist model of development. The Journal of Theoretical Biology 152:429–453.

[2] Reinitz J, Mjolsness E, Sharp DH (1995) Cooperative control of positional information in *Drosophila* by *bicoid* and maternal *hunchback*. The Journal of Experimental Zoology 271:47–56.

[3] Reinitz J, Sharp DH (1995) Mechanism of *eve* stripe formation. Mechanisms of Development 49:133–158.

[4] Jaeger J, Surkova S, Blagov M, Janssens H, Kosman D, et al. (2004) Dynamic control of positional information in the early *Drosophila* embryo. Nature 430:368–371.

[5] Jaeger J, Blagov M, Kosman D, Kozlov KN, Manu, et al. (2004) Dynamical analysis of regulatory interactions in the gap gene system of *Drosophila melanogaster*. Genetics 167:1721–1737.

[6] Tautz D, Lehmann R, Schnürch H, Schuh R, Seifert E, et al. (1987) Finger protein of novel structure encoded by *hunchback*, a second member of the gap class of *Drosophila* segmentation genes. Nature 327:383–389.

[7] Redemann N, Gaul U, Jäckle H (1988) Disruption of a putative Cys-zinc interaction eliminates the biological activity of the *Krüppel* finger protein. Nature 332:90–92.

[8] Nauber U, Pankratz MJ, Kienlin A, Seifert E, Klemm U, et al. (1988) Abdominal segmentation of the *Drosophila* embryo requires a hormone receptor-like protein encoded by the gap gene *knirps*. Nature 336:489–492.

[9] Mohler J, Eldon ED, Pirrotta V (1989) A novel spatial transcription pattern associated with the segmentation gene, *giant*, of *Drosophila*. The EMBO Journal 8:1539–1548.

[10] Shermoen AW, O'Farrell PH (1991) Progression of the cell cycle through mitosis leads to abortion of nascent transcripts. Cell 97:303–310.

[11] Foe VE, Alberts BM (1983) Studies of nuclear and cytoplasmic behaviour during the five mitotic cycles that precede gastrulation in *Drosophila* embryogenesis. The Journal of Cell Science 61:31–70.

[12] Gaul U, Jäckle H (1987) Pole region-dependent repression of the *Drosophila* gap gene *Krüppel* by maternal gene products. Cell 51:549–555.

[13] Eldon ED, Pirrotta V (1991) Interactions of the *Drosophila* gap gene *giant* with maternal and zygotic pattern-forming genes. Development 111:367–378.

[14] Kraut R, Levine M (1991) Spatial regulation of the gap gene *giant* during *Drosophila* development. Development 111:601–609.

[15] Jaeger J, Sharp DH, Reinitz J (2007) Known maternal gradients are not sufficient for the establishment of gap domains in *Drosophila melanogaster*. Mechanisms of Development 124:108–128.

[16] Surkova S, Kosman D, Kozlov K, Manu, Myasnikova E, et al. (2008) Characterization of the *Drosophila* segment determination morphome. Developmental Biology 313:844–862.

[17] Myasnikova E, Samsonova M, Kosman D, Reinitz J (2005) Removal of background signal from *in situ* data on the expression of segmentation genes in *Drosophila*. Development, Genes and Evolution 215:320–326.

[18] Houchmandzadeh B, Wieschaus E, Leibler S (2002) Establishment of developmental precision and proportions in the early *Drosophila* embryo. Nature 415:798–802.

[19] Mlodzik M, Gehring WJ (1987) Hierarchy of the genetic interactions that specify the anteroposterior segmentation pattern of the *Drosophila* embryo as monitored by *caudal* protein expression. Development 101:421–435.

[20] Brönner G, Jäckle H (1991) Control and function of terminal gap gene activity in the posterior pole region of the *Drosophila* embryo. Mechanisms of Development 35:205–211.

[21] Chu KW, Deng Y, Reinitz J (1999) Parallel simulated annealing by mixing of states. The Journal of Computational Physics 148:646–662.

[22] Chu KW (2001) Optimal Parallelization of Simulated Annealing by State Mixing. PhD Thesis, Department of Applied Mathematics and Statistics, Stony Brook University.

[23] Clyde DE, Corado MS, Wu X, Pare A, Papatsenko D, et al. (2003) A self-organizing system of repressor gradients establishes segmental complexity in *Drosophila*. Nature 426:849–853.

[24] Perkins TJ, Jaeger J, Reinitz J, Glass L (2006) Reverse engineering the gap gene network of *Drosophila melanogaster*. PLoS Computational Biology

2:e51. Epub 2006 May 19. http://compbiol.plosjournals.org/perlserv/?request=get-document&doi=10.1371/journal.pcbi.0020051.

[25] Ashyraliyev M, Jaeger J, Blom JG (2008) Parameter estimation and determinability analysis applied to *Drosophila* gap gene circuits. BMC Systems Biology 2:83.

[26] Weigel D, Jürgens G, Klingler M, Jäckle H (1990) Two gap genes mediate maternal terminal pattern information in *Drosophila*. Science 248:495–498.

[27] Pignoni F, Steingrimsson E, Lengyel JA (1992) *bicoid* and the terminal system activate *tailless* expression in the early *Drosophila* embyro. Development 115:239–251.
